# Transmission of SARS-CoV-2 among children and staff in German daycare centres

**DOI:** 10.1017/S0950268822001194

**Published:** 2022-07-08

**Authors:** Julika Loss, Juliane Wurm, Gianni Varnaccia, Anja Schienkiewitz, Helena Iwanowski, Anne-Kathrin Mareike Loer, Jennifer Allen, Barbara Wess, Angelika Schaffrath Rosario, Stefan Damerow, Tim Kuttig, Hanna Perlitz, Anselm Hornbacher, Bianca Finkel, Carolin Krause, Jan Wormsbächer, Anna Sandoni, Ulrike Kubisch, Kiara Eggers, Andreas Nitsche, Aleksandar Radonic, Kathrin Trappe, Oliver Drechsel, Kathleen Klaper, Andrea Franke, Antje Hüther, Udo Buchholz, Walter Haas, Lothar H. Wieler, Susanne Jordan

**Affiliations:** 1 Department of Epidemiology and Health Monitoring, Robert Koch Institute, Berlin, Germany; 2 Department of Infectious Disease Epidemiology, Robert Koch Institute, Berlin, Germany; 3 Centre for Biological Threats and Special Pathogens, Robert Koch Institute, Berlin, Germany; 4 Department of Methodology and Research Infrastructure, Robert Koch Institute, Berlin, Germany; 5 Leadership Robert Koch Institute, Robert Koch Institute, Berlin, Germany

**Keywords:** Children, daycare centres, SARS-CoV-2, secondary attack rate, transmission

## Abstract

In daycare centres, the close contact of children with other children and employees favours the transmission of infections. The majority of children <6 years attend daycare programmes in Germany, but the role of daycare centres in the SARS-CoV-2 pandemic is unclear. We investigated the transmission risk in daycare centres and the spread of SARS-CoV-2 to associated households. 30 daycare groups with at least one recent laboratory-confirmed SARS-CoV-2 case were enrolled in the study (10/2020–06/2021). Close contact persons within daycare and households were examined over a 12-day period (repeated SARS-CoV-2 PCR tests, genetic sequencing of viruses, symptom diary). Households were interviewed to gain comprehensive information on each outbreak. We determined primary cases for all daycare groups. The number of secondary cases varied considerably between daycare groups. The pooled secondary attack rate (SAR) across all 30 daycare centres was 9.6%. The SAR tended to be higher when the Alpha variant was detected (15.9% *vs.* 5.1% with evidence of wild type). The household SAR was 53.3%. Exposed daycare children were less likely to get infected with SARS-CoV-2 than employees (7.7% *vs.* 15.5%). Containment measures in daycare programmes are critical to reduce SARS-CoV-2 transmission, especially to avoid spread to associated households.

## Introduction

Since the beginning of the COVID-19 pandemic in March 2020, the role of daycare children in the spread of SARS-CoV-2 has been discussed controversially. In daycare centres children have close contact with each other and staff, possibly facilitating the transmission of SARS-CoV-2. Effective containment measures, such as physical distancing and wearing masks, are difficult to implement in early childhood. As of November 2021, COVID-19 vaccines have not been approved in Germany for application in toddlers and preschoolers yet. Given the high proportion of young children attending daycare (in Germany, 35% of 0- to 2-year-olds and 93% of 3- to 6-year-olds, [1]), understanding SARS-CoV-2 transmission within daycare centres is critical to inform adequate mitigation policies.

Only few studies have explored transmission dynamics in daycare centres or among children of kindergarten age. An early publication from Australia found that children were the primary case in only three of ten daycare centres with SARS-CoV-2 cases; in none of these daycare centres a secondary case was detected among close contacts [2]. According to a review by Spielberger *et al*., children with SARS-CoV-2 infected an average of 13.4% of their contacts [3], whereas more recent meta-analyses on SARS-CoV-2 report transmission rates of 4% and (approx.) 5% if children were registered as index case [4, 5]. These results cannot be transferred to daycare centres, since the analyses referred to broader age groups (0–9 years and older), and the data primarily stem from household studies, not from daycare programmes. A meta-analysis of contact tracing studies concluded that children are less susceptible than adults to SARS-CoV-2, but with large heterogeneity between studies [6]. The data basis on transmission risk of children in daycare centres is therefore still unsatisfactory. In addition, COVID-19 in childhood often goes along with no or only mild symptoms [7], which may obscure the role of children in transmission dynamics, as they may not be documented as index case in an outbreak. Transmission dynamics may also be influenced by virus variants. Increased transmissibility was postulated with the advent of SARS-CoV-2 ‘variants of concern’ (VOC), such as the Alpha or Delta variant [8, 9], although its role in outbreaks in daycare centres remains unknown. A recent study on the Omicron variant, which became dominant in Germany from January 2022 on, found a very high household transmission rate of 72% among index cases aged 0–4 years [10].

When SARS-CoV-2 began to spread in Germany in March 2020, several mitigation measures were implemented, such as physical distancing and hygiene measures. Daycare programmes were temporarily suspended, and restricted to a limited number of children (who had special needs or whose parents had critical jobs) from the beginning of 2021 on. Furthermore, a range of containment measures was officially recommended for daycare centres, i.e. hygiene plans, taking children's temperature or regular ventilation. Adherence to the recommended measures has been high. A large longitudinal study among ca. 8500 German daycare programmes showed that 92–100% of the participating centres had implemented regular ventilation and surface disinfection in autumn 2020, for example, and 87–92% of daycare were still maintaining these measures centres in May 2021 [11]. The staff used masks in contact with other staff members in 97% and 86% of daycare centres in winter 2020/21 and in June 2021, respectively [12, 13]. Official COVID-19 guidelines for daycare programmes also concede that physical closeness and contact is important when interacting with toddlers and pre-schoolers, and could – and should – not be suspended in the day-to-day life of a daycare centre even during a pandemic. Measures such as physical distancing can therefore only be implemented to a limited extent in daycare programmes [14]. The study presented in this paper was carried out during the ‘second wave’ (10/20–02/21) and ‘third wave’ (03/21–05/21) of the pandemic in Germany, during which numerous outbreaks occurred in daycare centres (between ca. 50–200 per week nationwide) [15]. During that period, vaccinations became available to only certain groups of the population in Germany (e.g. for staff of daycare centres since February 2021 [12]), but were not available for children. Due to the timing of this study, it was not possible to investigate the role of more recent variants of concern, like Delta or Omicron, as those emerged later in Germany [13].

In order to gain a better understanding about the transmission risk of SARS-CoV-2 within daycare centres and corresponding households of infected study participants, we conducted an observational study in German daycare centres with at least one notified case of SARS-CoV-2 (‘Corona outbreak-related examinations in daycare centres’, COALA). The aim of the study was to identify secondary infections among exposed children and adults in daycare centres and their corresponding households, the primary endpoint being the secondary attack rate (SAR) in both settings.

## Methods

### Study design

Design and methods of COALA are described in detail in a study protocol [16]. We chose a case-ascertained study design with longitudinal collection of data and specimen. We included daycare centre groups in which one or more SARS-CoV-2 positive cases (child or staff) were detected, and parents and staff consented to participate. From October 2020 till June 2021, 30 daycare groups from 20 different communities all over Germany were included in the study. SARS-CoV-2 cases as well as their close contacts within the respective daycare group and household were examined over a 12-day period, including the collection of biological specimens and the documentation of symptoms. In order to reconstruct transmission chains and identify primary cases, clinical symptoms and possible exposition towards the virus were assessed retrospectively through questionnaires. The SARS-CoV-2-antibody status was assessed in capillary blood samples to detect previous infections.

### Recruitment

Information about newly diagnosed SARS-CoV-2 cases in daycare centres was gathered through collaboration with several local health authorities or contacts with (umbrella) associations that run daycare facilities. The recruitment was restricted to daycare centres in which the participants could be seen and tested by the study personnel within 4–6 days after the PCR test of the index case; it also followed a purposive sampling strategy, to allow for a roughly equal distribution of children and adults among index cases. Index cases and their close contacts (within the daycare group) were asked to participate, both children and adults. Household members of index cases and infected close contacts were included as well. Written informed consent was obtained from each participant. Participants received a monetary incentive, in order to increase adherence to the complex and time-consuming self-sampling schedule.

### Procedure of collecting data and biological samples

**Fig. 1.**
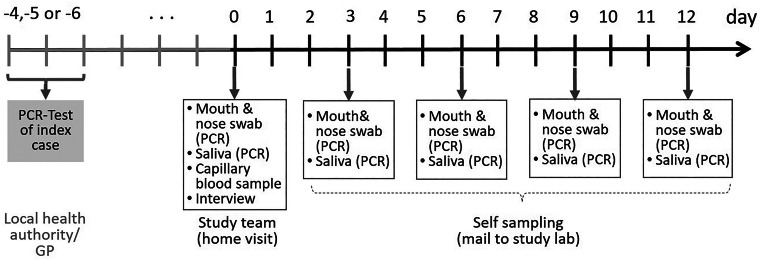
Time schedule for bio samples taken from the participants of the COALA study (SARS-CoV-2 index cases, secondary cases and close contacts of SARS-CoV-2 cases in the respective daycare centre group and households). Participants were enrolled 4–6 days after the index case got tested. GP, general practitioner.

As study participants were isolated or quarantined, they were visited at home by trained study personnel within four to six days after the PCR test of the index case. This timeframe was chosen based on the mean incubation time of SARS-CoV-2 [17], and in order to ensure the detection of secondary cases. We took combined mouth and nose swabs, saliva samples and capillary blood samples on dried blood spot cards from each participant. Participants were also instructed to take combined mouth and nose swabs and saliva samples from themselves and/or their children in a cycle of three days (over a total period of 12 days) as well as to return their samples independently to the laboratory via mail (Fig. 1). Mouth and nose swabs and saliva samples were chosen instead of nasopharyngeal swabs because they are more suitable for self-testing and testing of children, respectively, while presenting convincing sensitivity and specificity [18–20].

### Laboratory testing

Combined mouth and nose swabs and saliva samples were tested for viral RNA of SARS-CoV-2 by real-time reverse-transcriptase-polymerase chain reaction (rRT-PCR). Positive samples were also screened for the Alpha variant by PCR, and the genome was sequenced to detect virus lineages [21]. Sequencing was done on Illumina ISeq (2 × 150 bp; CleanPlex amplification) or ONT MinION devices (ARTIC v3 amplification [22]). Virus sequences were determined using covpipe (Illumina data, [23]) or poreCov (MinION data, [24]). Capillary blood samples were tested for IgG antibodies against the S1 domain of SARS-CoV-2 spike protein with a semiquantitative Enzyme-Linked Immunosorbent Assay (ELISA from Euroimmun, Germany).

### Questionnaires

Standardised telephone interviews were conducted with each participating household, to gain information on each household member regarding a previous SARS-CoV-2 infection, SARS-CoV-2 exposition, history of COVID-19 symptoms and the children's attendance times at the daycare centre. Further information on the outbreak was gained through interviews with the respective local health authorities and daycare director. These interviews included, among others, questions on implemented containment measures, such as frequency of ventilation and disinfection or use of masks and general characteristics of the daycare centre, such as size of used rooms.

### Definitions

All participants from the included daycare groups (SARS-CoV-2 cases and close contacts) constituted the daycare centre cohort. The household cohort comprised the secondary cases of the daycare group (children or staff members) and their respective household members. The index case was defined as the first case of SARS-CoV-2 to be reported to the local health authority, whereas the primary case was considered to be the origin of the infectious event in the daycare group or household. Primary case and index case were not necessarily identical with each other. A secondary case was defined as a close contact of the primary case who tested positive in a SARS-CoV-2 PCR test up to 9 days after the household visit. Close contacts were defined as individuals exposed to the confirmed SARS-CoV-2 cases within the daycare group or household, respectively (<1.5 m for more than 15 min). Secondary cases infected in the daycare centre were considered to be primary cases in their respective households. The secondary attack rate (SAR) is the proportion of infected contacts out of the total number of (susceptible) contacts within a setting.

### Determination of primary cases

Based on the results of the laboratory testing and of the standardised interviews, we reconstructed infection pathways in each household and daycare group, and thereby determined the probable primary cases. The probable date of infection with SARS-CoV-2 was estimated for every participant with SARS-CoV-2 following the proceedings suggested by Layan *et al*. [25], considering (a) information on symptom onset and (b) date of first positive PCR test. Furthermore, we considered information on exposure to SARS-CoV-2 and IgG-antibody status against SARS-CoV-2.

Probable primary cases were determined for all investigated daycare groups. In four daycare groups, there was convincing evidence that an individual other than the index case had been the first person to be infected within the group, mainly due to earlier onset of symptoms or exposition to an earlier laboratory-confirmed COVID-19 infection in the household or both. In two of those four daycare groups, a child was determined as the probable primary case instead of a reported adult index case. There was also a slight change in the number of close contacts (plus two), as in two daycare groups where two children (siblings)/staff had been registered as simultaneous index cases, another child was determined as the probable primary case.

### Statistical analyses

Descriptive statistics were performed to describe secondary cases and secondary attack rates. Confidence intervals were calculated on the logit scale using robust standard errors, accounting for the clustering within daycare groups or households, using the Stata survey command. Odds ratios (OR) and *P*-values were on a GEE (generalised estimating equations) logistic regression model [26] with exchangeable working correlation to account for clustering. A Mancl-de Rouen correction was applied [26–29]. All analyses were performed using Stata 17.0.

### Sample characteristics

Between October 2020 and June 2021, 85 daycare centres with an acute SARS-CoV-2 case were reported to the study team. Out of 85 these, 30 daycare groups were included in the COALA study. Reasons for not including a daycare centre were a lack of research capacity (i.e. in case of a high number of simultaneously reported outbreaks), the insufficient response among potential participants or prioritisation of daycare centres with a child as an index case. In 17 of the 30 daycare groups, the index case was reported to be a child (or two children, who were siblings and tested positive on the same day, *n* = 3), in 13 daycare groups it was a staff member.

Overall, 282 daycare children (1–6 years), 91 staff members (19–68 years) and 45 household members (1–69 years) of secondary cases in the daycare groups were included in this analysis (Table 1). In most daycare groups, not all members of the included daycare groups consented to participate in the study; the response rate among index cases was 74%, among close contacts 60% (children) and 57% (staff), respectively. IgG antibodies against SARS-CoV-2 were detected in 22 cases (children: *n* = 8, staff: *n* = 12; adults in households *n* = 2); 6 had a simultaneous positive PCR-test and no history of prior infection, and were determined to be in a state of fresh seroconversion (3 of them were primary cases in the daycare group).

**Table 1.** Characteristics of the study population in daycare groups and households with at least one acute laboratory-confirmed SARS-CoV-2 case, COALA study, Germany, 10/2020–06/2021

	Children and adults (staff) in the daycare groups with at least one SARS-CoV-2 case			Household members of the secondary cases in the daycare groups		
	Children	Adults	Total	Children	Adults	Total
Sex						
Female	128	82	210	8	17	25
Male	154	9	163	3	17	20
Age						
1–6 years	282	–	282	2	–	2
7–17 years	–	–	–	9	–	9
18+ years	–	91	91	–	34	34
Role						
Primary case^a^	16	7	23	–	–	–
Close contact^b^	266	84	350	11	34	45
Total	282	91	373	11	34	45

a In 7 daycare groups, the primary cases were tested for SARS-CoV-2 and officially confirmed by the local health authorities, but did not participate in our study. Nevertheless, these daycare groups were included, as a substantial number of close contacts consented to participate, which was essential for calculating the secondary attack rate.

b Including 7 siblings of primary cases in the daycare group that are excluded in further analysis because it cannot be determined whether they got infected in the daycare group or their household.

## Results

### Secondary transmissions in daycare groups

We detected 33 secondary SARS-CoV-2-cases during the course of the study through PCR testing (Tables 2 and 3); 3/33 were not detected before the self-sampling phase. 6 additional secondary cases were close relatives (e.g. siblings) of the primary cases who attended the same daycare group; they were excluded from the analysis, as it could not be determined whether they got infected during daycare or at home. The number of secondary transmissions varied considerably among daycare groups. In the majority of the daycare groups, no secondary cases (22/30) or only 1–2 secondary cases (3/30) were detected among the participating close contacts. The maximum number of secondary attacks observed in one daycare group was 11. When comparing daycare centres with and without secondary cases, we did not find any significant differences in terms of containment measures and other characteristics of the daycare centre. Daycare groups with no secondary cases among the participants tended to have more space per person, and to spend longer hours outdoors, than those with one or more secondary cases, but these differences were not significant (Supplementary material, Table S5).

**Table 2.** Characteristics of the daycare groups with at least one acute laboratory-confirmed SARS-CoV-2 case, COALA study, Germany, 10/2020–06/2021

Daycare group	Month of outbreak	Type of primary case	Number of close contacts	Number of participating close contacts^a^	Number of secondary transmissions	Secondary attack rate	Number of close contacts with existing antibodies at day 0	Virus variant
1	10/20	Child	18	13	0	0.0	0	Wildtype/non-VOC
2	10/20	Adult	Unknown	9	1	11.1	0	Wildtype/non-VOC
3	11/20	Adult	Unknown	18	0	0.0	0	Wildtype/non-VOC
4	11/20	Child	20	14	0	0.0	0	Wildtype/non-VOC
5	12/20	Child	Unknown	12	0	0.0	0	Wildtype/non-VOC
6	01/21	Adult	32	23	0	0.0	1	Unknown
7	01/21	Child	16	13	1	7.7	0	Wildtype/non-VOC
8	01/21	Child	Unknown	7	0	0.0	0	Unknown
9	01/21	Adult	10	8	0	0.0	1	Wildtype/non-VOC
10	02/21	Adult^b^	17	12	3	25.0	0	Wildtype/non-VOC
11	02/21	Child^b,c^	Unknown	11	4	36.4	0	VOC-Alpha
12	02/21	Child	16	9	0	0.0	0	VOC-Alpha
13	02/21	Adult	12	10	0	0.0	0	VOC-Alpha
14	02/21	Child	16	5	0	0.0	1	Wildtype/non-VOC
15	02/21	Child	Unknown	15	0	0.0	0	VOC-Alpha
16	02/21	Child	27	18	0	0.0	0	VOC-Alpha
17	03/21	Child	20	7	0	0.0	0	VOC-Alpha
18	03/21	Adult	12	7	0	0.0	0	Unknown
19	03/21	Child	25	16	0	0.0	1	VOC-Alpha
20	03/21	Adult	23	13	1	7.7	0	Wildtype/non-VOC
21	03/21	Child	15	9	0	0.0	0	Unknown
22	03/21	Child	26	8	0	0.0	1	VOC-Alpha
23	03/21	Adult	23	14	4	28.6	0	VOC-Alpha
24	03/21	Child^b,c^	30	14	11	78.6	1^d^	VOC-Alpha
25	04/21	Child	Unknown	7	0	0.0	2	VOC-Alpha
26	04/21	Child^b^	29	19	8	42.1	1^d^	VOC-Alpha
27	05/21	Adult	28	7	0	0.0	1	VOC-Alpha
28	05/21	Adult	12	7	0	0.0	3	VOC-Alpha
29	06/21	Child	21	10	0	0.0	1	Unknown
30	06/21	Child	Unknown	8	0	0.0	3	VOC-Alpha
Total	–	–	448	343	33	9.6^e^	17	–

^a^7 siblings of primary cases were excluded (6 of them were tested positive).

^b^The primary case was not the index case.

^c^The index case was an adult, the primary case a child.

^d^The person was also tested positive on SARS-CoV-2.

^e^Pooled secondary attack rate (33 of 343 participating close contacts were tested positive).

**Table 3.** Close contacts and secondary cases in daycare groups with at least one acute laboratory-confirmed SARS-CoV-2 case by outbreak specific characteristics, COALA study, Germany, 10/2020–06/2021

Outbreak specific characteristics	Number of daycare groups	Number of close contacts			Number of secondary cases		
		Children	Adults	Total	Children	Adults	Total
Type of primary case							
Child	19	162	53	215	14	10	24
Adult (staff)	11	97	31	128	6	3	9
Type of close contacts							
Children	30	259	–	259	20	–	20
Adults (staff)	30	–	84	84	–	13	13
Virus variant in daycare group							
Wild-type/non-VOC	10	86	31	117	2	4	6
Alpha variant	15	130	40	170	18	9	27
Unknown	5	43	13	56	0	0	0
Total	30	259	84	343	20	13	33

### Secondary attack rate in daycare groups

When the results of all daycare groups were pooled, the mean SAR in the daycare cohort was estimated at 9.6% (95% CI 4.0–21.3%) (Table 4). When close contacts with existing SARS-CoV-2 antibodies (*n* = 17) were excluded from the calculation due to an assumed lower susceptibility, the attack rate remained similar (9.5%, 95% CI 4.1–20.6%). The transmission risk from paediatric primary cases did not differ significantly from that from adult primary cases (11.2% *vs.* 7.0%, *P* = 0.706). When excluding a daycare group with extraordinarily high transmission of 11 secondary cases (no. 24), the estimation of the SAR from children decreased from 11.2 to 6.5% (95% CI 1.7–21.5%).

**Table 4.** Secondary attack rates in daycare groups with at least one acute laboratory-confirmed SARS-CoV-2 case by outbreak specific characteristics, COALA study, Germany, 10/2020–06/2021

Outbreak specific characteristics	Secondary attack rate (95% CI)^a^	Unadjusted		Adjusted^a^	
		Odds ratio (95% CI)	*P*-value	Odds ratio (95% CI)	*P*-value
Type of primary case					
Child	11.2 (3.6–29.7)	1.00		1.00	
Adult (staff)	7.0 (2.5–18.5)	0.73 (0.13–4.01)	0.706	0.79 (0.13–4.82)	0.794
Type of close contacts					
Children	7.7 (2.7–19.9)	1.00		1.00	
Adults (staff)	15.5 (6.4–33.0)	**3.03** (1.45–6.32)	0.005	**3.11** (1.43–6.77)	0.006
Virus variant in daycare group					
Wild type/non-VOC	5.1 (1.8–13.7)	1.00		1.00	
Alpha variant	15.9 (5.9–36.1)	2.72 (0.52–14.4)	0.225	1.97 (0.36–10.7)	0.412
Total^b^	9.6 (4.0–21.3)	–	–	–	–

a Adjusted for the variables in the first column.

b The working correlation in the GEE model (intercept only) was estimated as 0.42 (excluding daycare group 24: 0.26). In the fully adjusted model, the working correlation was 0.36 (excluding daycare group 24: 0.22).

Most studies do not determine primary cases, but calculate the SAR based on the reported index cases. To investigate whether the determination of primary cases made a difference to the SAR, we also estimated it based on index cases. In this case, the result for the transmission risk from infected children to close contacts would have been different: 4.2% (95% CI 0.7–20.6%) instead of 11.2%, *vs.* 17.3% (95% CI 6.2–39.9%) instead of 7.0% from adult index cases (staff) (*P* = 0.095). This could be explained by the fact that those two daycare groups where the determined primary case was a child, whereas the index case reported to the health authority had been an adult, were no. 11 and no. 24. These were two outbreaks with a relatively high number of secondary cases (*n* = 4 and *n* = 11).

In daycare groups with confirmed Alpha variant infection(s) (*n* = 15), the estimated SAR was higher than the SAR for groups with confirmed infections of the wild-type or another non-VOC (*n* = 10; 5.1% *vs.* 15.9%), but not statistically significant. Regarding susceptibility towards the virus, children in the daycare groups were less likely to have contracted SARS-CoV-2 than staff. 7.7% of all participating children who were exposed to SARS-CoV-2 within their daycare group tested positive for SARS-CoV-2, compared to 15.5% of all adult close contacts (*P* = 0.005).

### Secondary attack rate in households

Those children (*n* = 20) and staff members (*n* = 13) who got infected with SARS-CoV-2 during daycare lived in 24 households with 45 close contacts (34 adults, mostly parents and 11 children, mostly siblings). From those, two single-person households of infected staff members were consecutively excluded from further analysis. In six of the remaining 22 households, two or more infected daycare children or staff members lived together in one household. Overall, secondary infections among further household members were observed in 12 of the 22 households (54.5%). In total, 24 of all 45 close contacts in households were diagnosed as secondary SARS-CoV-2 cases, resulting in a mean household SAR of 53.3% (35.4–70.4%), the SAR being significantly higher in households than in the daycare setting (*P* = 0.000).

## Discussion

### Summary of main findings

Across the 30 studied daycare centres with a SARS-CoV-2 case, the transmission risk was very heterogeneous. In most daycare groups, there were no transmissions among participants, whereas there were few daycare groups in which we detected numerous secondary cases. When pooling the daycare groups, we found a secondary attack rate of 9.6%, which was significantly lower than the SAR in the associated households (53.3%). A higher proportion of infections among close contacts was found in daycare groups with the Alpha variant, as compared to wild-type or non-VOC (15.9% *vs.* 5.1%, n.s.). There was no significant difference in the secondary attack rate when children were the primary case in comparison to when adults were the primary case, but exposed adults had a significantly higher risk of contracting SARS-CoV-2 than children. In case of an infection in the daycare group, the virus was introduced to 54.5% of corresponding households. A comparison between daycare centres with and without SARS-CoV-2 infections did not show any significant differences regarding containment measures or other characteristics of the daycare centres.

### Comparison with other studies

A comparable study was conducted in one federal state of Germany, using data from routine contact tracing. It included 99 index cases from daycare centres and 4392 contacts in daycare [30], with a resulting SAR of 2.5%. As the study was performed earlier (from August to December 2020), it could not include cases with the Alpha variant, which may account for the lower SAR in this study as compared to our analysis (9.6%); the SAR of non-VOC outbreaks in our study was 5.1%. The follow-up period of 12 days after enrolment and regular testing could identify additional cases in COALA, who otherwise might have not been detected in routine contact tracing due to mild or absent symptoms, which could also explain the higher SAR found in our study. In our study, there were many enrolled daycare groups in which no secondary cases were identified among participants, whereas some daycare groups presented with 4 up to 11 secondary cases. Heterogeneity among educational facilities where exposure to SARS-CoV-2 occurred has also been observed in a study conducted in New South Wales, Australia [2]. In a large study on household transmission in England from summer 2020 [31], the secondary attack rate was 5.5 secondary cases per 100 household contacts. This is comparably lower than the SAR found in our study (53%). The difference is likely to reflect a difference in the data collection approach, i.e. passive surveillance *vs.* the whole-household testing in our study. Additionally, the data collection in the study of Hall *et al*. took place before the dominance of the Alpha variant, which may also explain the lower SAR. Similar to our findings, Hall *et al*. showed that children were less likely to become infected than adults.

### Interpretation of results and implications for policy, practice and research

Our results thus confirm that SARS-CoV-2 transmissions occur in daycare centre groups, and that both children and staff members can play a role in infectious events. There was a high degree of heterogeneity of transmission rates across the included daycare groups. This is not an unusual phenomenon, as the mandatory reporting data of outbreaks in daycare centres in Germany from the same period show [32]. We can only speculate about the reasons, as we could not find significant differences between those groups with and without secondary cases. It is well known that SARS-CoV-2 transmission is highly overdispersed [33]. For one, the virus variant may play a role. SAR was considerably higher in those daycare groups were the Alpha variant was identified, although not statistically significant (which can also have been influenced by the small sample size). Local factors may have also facilitated the transmission, such as aerosol-promoting conditions or a closer and longer contact among children and/or staff members. In those daycare groups with secondary cases (*n* = 8), there was a tendency of less space per person and more hours spent indoors, which may be worth investigating in larger samples.

The predominance of the Alpha variant resulted in a higher transmission risk in daycare (although statistically not significant). This shows that the role of children, or daycare centres, may change considerably over the course of the pandemic, depending on the predominant genetic lineage. This assumption is corroborated by studies on the Omicron variant, which has been dominant in Germany and other countries worldwide since January 2022, and for which an even higher transmission rate of 72% of young children (aged 0–4 years) was described in a household study in the USA [10]. This confirms the need for continuous monitoring of outbreaks and transmission patterns in this setting. When comparing transmission rates from different studies, it is also important to take into account the method of data collection. Outbreak investigations with intensive follow-up testing may result in the detection of more secondary cases, and therefore a higher SAR, as compared to passive reporting of cases, which may tend to under-estimated transmission risk [31]. Further studies should compare different methodologies that are used to analyse transmission risk.

Still, given the fact that physical distancing and wearing of masks is hardly possible among toddlers and preschoolers within a daycare centre group, it is striking that the SAR in this setting is still significantly lower than in households (9.6% *vs.* 53.3%). Even if only a relatively small percentage of close contacts gets infected with SARS-CoV-2 in the daycare group, those who were infected were quite probable to spread the infection to their families and household members. Daycare centres may have the potential to serve as bridges over which the virus can be spread to other households. Daycare providers can therefore help slow the spread of COVID-19 by implementing mitigation measures in their programmes. One of the most effective approaches is keeping the groups within daycare centres small and consistent, and minimising contact between these groups [11]. The role of different COVID-19 testing strategies is still to be evaluated.

When analysing the SAR within a specific setting, or from a specific population (e.g. children), it is worth determining the primary case where possible, even when analysing pooled samples. Otherwise, the role of children in outbreaks may be underestimated.

### Strengths and limitations

The study is subject to some limitations. First, the sample size is relatively small, which is why the results show a relatively high statistical uncertainty. Due to the small sample size, only large differences between groups can be detected. On the other hand, our sample, underwent a very elaborate and comprehensive investigation, with extensive retrospective and prospective data collection and various laboratory tests. Studies with considerably larger samples mostly rely on routine data obtained by public health offices, which may sometimes lack the thoroughness of examination and richness of detailed data, thereby possibly missing out on some later secondary cases or the detection of asymptomatic primary cases, which is a strength of our study. Furthermore, the sampling of daycare centres was not random and might not be representative. In addition, with the study being voluntary, the participation among close contacts in daycare groups and households was mostly not complete. We cannot rule out a selection bias, for example, severely ill persons may have declined more frequently than others the invitation to participate.

When determining the probable primary case, we were sometimes also restricted by the fact that not all daycare group members participated. Therefore, we could not rule out that maybe the real primary case would be found outside of our sample. Including additional data from local health authorities and daycare directors was meant to compensate for this drawback.

A strength of our study is that we were largely successful in determining primary cases. The reconstruction of the transmission chain in each participating daycare group yielded probable primary cases which differed from the registered index cases in four of the daycare groups, twice with a change from staff member to child, thereby producing a different SAR when stratified for child *vs.* adult.

The prospective design was a strength of the study, as it enabled us to detect additional secondary cases which might have not been noticed if the investigation had taken place only briefly after the diagnosis of the index case. A further strength is the richness of data, including measurement of antibodies and close monitoring of symptoms, which is unusual in the non-clinical setting, and helped us track transmission dynamics and determine primary cases. By determining probable primary cases, we decreased the risk that children with mild or no symptoms may be overlooked as an infectious source in the daycare centres. Genomic sequencing of SARS-CoV-2 was a further advantage of the study, as the existence of a VOC (Alpha), which was found in 50% of included daycare group outbreaks, could partly explain the heterogeneity in the number of secondary cases between daycare groups. There was a lack of (sufficient) material to perform genomic sequencing in five daycare groups, however, which was favoured by the fact that no or few participants were tested positive, so we cannot rule out that the SAR for daycare groups with the Alpha variant was overestimated.

A strength of the analysis is that the clustering of contacts within daycare groups and households, respectively, was taken into account when calculating confidence intervals and *P*-values, and that the effect of heterogeneity in SAR estimates across daycare groups on the results was explored.

## Conclusion

The COALA study adds to the global literature on SARS-CoV-2 by providing evidence on virus transmission in the daycare centre setting, which has been understudied so far. Studies may risk to underestimate the secondary attack rate of children if they refer to reported index cases instead of determining probable primary cases or implement only short follow-up periods. The study also shows that defining the SAR of SARS-CoV-2 cases in children is a moving target, as it may change over time with novel genetic variants of the virus and with varying range of mitigation measures that are put into place in daycare centres. Therefore, continuous monitoring of outbreaks and transmission patterns in daycare centres can contribute to developing, implementing and adapting adequate prevention and mitigation measures. Reducing transmission in daycare centres is critical, as children and staff members who got infected in the daycare group have a high risk of spreading the virus to their households.

## Data Availability

The authors confirm that some access restrictions apply to the data underlying the findings. The data set cannot be made publicly available because informed consent from study participants did not cover public deposition of data. However, the minimal data set underlying the findings is archived in the ‘Health Monitoring’ Research Data Centre at the Robert Koch Institute (RKI) and can be accessed by researchers on reasonable request. On-site access to the data set is possible at the Secure Data Centre of the RKI‘s ’Health Monitoring' Research Data Centre. Requests should be submitted to the ‘Health Monitoring’ Research Data Centre, Robert Koch Institute, Berlin, Germany (e-mail: fdz@rki.de). Genome sequences determined within this study are published in ENA/GISAID ‘StudyID’.

## Supplementary material

For supplementary material accompanying this paper visit https://doi.org/10.1017/S0950268822001194. click here to view supplementary material

## Data access

Genome sequences determined within this study are published in ENA/GISAID ‘StudyID’.
