# Supplementary material for: Transmission of SARS-CoV-2 among children and staff in German daycare centres
Source: Epidemiol Infect. 2022 Jul 8;150:e141. doi: 10.1017/S0950268822001194 (PMC9343344; doi:10.1017/S0950268822001194)
Supplement: Supplementary file 1 [file S0950268822001194sup001.docx]

**Supplementary material**

Table 5: Group and setting characteristics as well as containment measures of included daycare centres: comparison between centres with and without secondary cases. Differences are not statistically significant.

| **Characteristics of the outbreak/daycare centre** | **Daycare centres without SARS-CoV-2 transmission (n=22)** | **Daycare centres with SARS-CoV-2 transmission (n=8)** |
| --- | --- | --- |
| **Mean age of children (years)** | 3.6 | 3.8 |
| **Symptoms of the primary case**  Asymptomatic primary case  Pre-symptomatic primary case  Symptomatic primary case  Not known | 14 % (3)  36% (8)  27% (6)  23% (5) | 12,5% (1)  25% (2)  25% (2)  37,5% (3) |
| **Rooms/spaces**  Size of the room in which contact persons were taken care of (mean sqm values)  Square meter per contact person | 75.6  4.5 | 52.4  2.2 |
| **Hours/day spent outside**  < 2  > 2  Not known | 55% (12)  36% (8)  9% (2) | 75% (6)  12.5% (1)  12.5% (1) |
| **Hygiene measures** | | |
| **Disinfection of surfaces**  Multiple times a day  Less than multiple times a day | 23% (5)  77% (17) | 32.5% (3)  62.5% (5) |
| **Ventilation (opening windows)**  Yes  No  Number/day (mean) | 100% (22)  0%  10 | 87% (7)  13% (1)  14 |
| **Staff wearing masks among each other**  Never/rarely/sometimes  Often/always  Not known  **Staff wearing masks in contact with children**  Never/rarely/sometimes  Often/always  Not known | 36% (8)  64% (14)  0%  73% (16)  27% (6)  0% | 25% (2)  63% (5)  13% (1)  75% (6)  13% (1)  13% (1) |
